# Nuclear matrix associated RNA datasets of posterior silk glands of *Bombyx mori* during 5th instar larval development

**DOI:** 10.1186/s13104-021-05872-6

**Published:** 2021-12-18

**Authors:** Alekhya Rani Chunduri, Anugata Lima, Resma Rajan, Anitha Mamillapalli

**Affiliations:** Department of Biotechnology, Institute of Science, GITAM (Deemed To Be University), Visakhapatnam, 530 045 India

**Keywords:** Nuclear matrix, RNA, *Bombyx mori*, Posterior silk glands, Development, Objective

## Abstract

**Objectives:**

*Bombyx mori* is the key contributor to industrial silk production. The maximum production of silk occurs during 5th instar. The posterior silk glands in the larvae are responsible for the production of the main component of silk fibre—fibroin. The expression of genes and their regulation are dependent on the chromatin architecture. The nuclear matrix supports its structure and function by anchoring specific regions to regulate gene expression. The major constituent of the nuclear matrix, crucial to its structural and temporal maintenance, is its RNA. Therefore, the study of nuclear matrix RNA of the posterior silk glands on different days of 5th instar larval development is essential to understand its association to differential expression of genes.

**Data description:**

The tissue-specific developmental association of nuclear matrix RNA (NuMat RNA) at the genome level has not been done so far for any organism. *Bombyx mori*, CSR2 X CSR4 is the most popular dihybrid strain in India. The nuclear matrix RNA was isolated from day 1, day 5 and day 7 of 5th instar posterior silk glands of *Bombyx mori.* The NuMat RNA was sequenced using Illumina platform. The reads obtained were processed and the datasets were deposited in NCBI.

## Objective

*Bombyx mori* (*B. mori*) is the most economically beneficial contributor to sericulture. While silk production was traditionally limited to textiles and crafts, the recent innovative discovery of its applications such as those in medicine and cosmetology has broken barriers of its importance and paved way to research aimed to improve the parameters of silk quality and quantity to cater to its wide range of uses. The silk fibre consists of fibroin, its main component and sericin, which binds the fibroin together to form the silk thread. Fibroin is produced by the posterior silk glands. Gene expression is linked to the spatial and temporal organization of the chromatin facilitated by their anchoring to the nuclear matrix.

The nuclear matrix (NuMat) is the non-chromatin residual nuclear structure, which remains following nuclease treatment and salt extraction of isolated nuclei. Although, it consists of DNA, RNA, and protein, the RNA component is considered its major constituent [1–4] which is evidenced by its high sensitivity to RNase treatment [5]. As the NuMat RNA is the main contributor to the structural stability of the nuclear matrix, we aimed to study its changes and dynamics exhibited on different days (day 1, day 5 and day 7) in posterior silk glands during 5th instar larval development of *B. mori* larvae. This study explores first time the developmental datasets of NuMat RNA in posterior silk glands which can be used to understand the possible role in regulation of gene expression. It lays a foundation to further research to embark new avenues in advancement of sericulture. This data was generated as a part of a study to understand the developmental dynamics associated with the nuclear matrix associated RNA in the posterior silk glands of 5th instar larvae of *B. mori.*

## Data description

### Nuclear matrix and RNA isolation

The double hybrid, Bivoltine, CSR2 X CSR4 variety of 4th moult *B. mori* larvae were collected from the Department of Sericulture, Srikakulam, Government of Andhra Pradesh. Fresh mulberry leaves (V1 variety) were used to feed larvae throughout the 5th instar stage. Posterior silk glands (PSGs) were dissected from 5th instar larvae on day 1, day 5, and day 7 under sterile conditions. PSGs from a single rearing were pooled and homogenized on all three days in nuclear isolation buffer (5 times volume of the weight of the tissue) and processed for nuclei and nuclear matrix isolation by following the standard protocol for isolation through nuclease digestion and salt extraction [6]. The nuclear and nuclear matrix pellets were then used for RNA isolation with TRIzol reagent [7].

### Library preparation and sequencing

RNA sequencing libraries were prepared with Illumina-compatible NEBNext® Ultra™ II Directional RNA Library Prep Kit (New England BioLabs, MA, USA) at Genotypic Technology Pvt. Ltd., Bangalore, India. 500 ng of total RNA was taken for mRNA isolation, fragmentation and priming. Fragmented and primed mRNA was further subjected to first strand synthesis followed by second strand synthesis. The double stranded cDNA was purified using JetSeq Beads (Bioline, Cat # BIO-68031). Purified cDNA was end-repaired, adenylated and ligated to Illumina multiplex barcode adapters as per NEBNext® Ultra™ II Directional RNA Library Prep protocol followed by second strand excision using USER enzyme at 37˚C for 15 min. Illumina Universal Adapters used in the study were: 5ʹ-AATGATACGGCGACCACCGAGATCTACACTCTTTCCCTACACGACGCTCTTCCGATCT-3ʹ and Index Adapter: 5ʹ-GATCGGAAGAGCACACGTCTGAACTCCAGTCAC [INDEX] ATCTCGTATGCCGTCTTCTGCTTG-3ʹ. Adapter ligated cDNA was purified using JetSeq Beads and was subjected to 8 cycles for Indexing- (98 °C for 30 s, cycling (98 °C for 10 s, 65 °C for 75 s) and 65 °C for 5 min) to enrich the adapter-ligated fragments. Final PCR products (sequencing libraries) were purified with JetSeq Beads, followed by library quality control check. Illumina-compatible sequencing libraries were quantified by Qubit fluorometer (Thermo Fisher Scientific, MA, USA) and fragment size distribution analysis was carried out on Agilent 2200 TapeStation. The libraries were sequenced on Illumina HiSeq X Ten sequencer (Illumina, San Diego, USA) using 150 bp paired-end chemistry following manufacturer’s procedure.

### Downstream processing and analysis of data

The raw sequenced data obtained from Illumina sequencing platform as paired-end reads were labelled SG 1, SG 5 and SG 7 (day 1, day 5 and day 7) respectively (Datasets 1, 2 and 3) and the quality control for each of these three datasets was carried out using FastQC tool v1.1 [8]. The reads were processed to obtain high quality reads (Data file 1). The removal of the adaptor sequences and low quality bases was carried out using the ‘Trim Galore!’ tool. Bowtie2 was used to align the datasets to the reference genome [9] with the default parameters. This mapped data was further used for downstream analysis. SSR prediction was carried out with the mapped datasets (SG 1, SG 5 and SG 7) using the MISA software. Gene identification was also performed against the mapped data (Data file 2). The downstream processing and analysis of the data were performed as a part of the study of developmental dynamics of nuclear matrix associated RNA in 5th instar posterior silk glands of *B. mori.* The datasets and data from the analysis are provided in Table 1.

**Table 1:** Overview of data files/data sets

Label	Name of data file/data set	File types (file extension)	Data repository and identifier (DOI or accession number)
Data set 1	SG 1 dataset (5th instar day 1 PSG NuMat RNA)	.fastq	NCBI Sequence Read Archive, https://identifiers.org/ncbi/insdc.sra:SRX9472457 [10]
Data set 2	SG 5 dataset (5th instar day 5 PSG NuMat RNA)	.fastq	NCBI Sequence Read Archive, https://identifiers.org/ncbi/insdc.sra:SRX7730613 [11]
Data set 3	SG 7 dataset (5th instar day 7 PSG NuMat RNA)	.fastq	NCBI Sequence Read Archive, https://identifiers.org/ncbi/insdc.sra:SRX9495290 [12]
Data file 1	Total Reads Vs. Processed reads	.pdf	figshare, https://doi.org/10.6084/m9.figshare.16760422.v1 [13]
Data file 2	Statistics of sequenced reads, SSRs and expression data from datasets	.pdf	figshare, https://doi.org/10.6084/m9.figshare.16569543.v2 [14]

## Limitations

The data stated in this paper was obtained from a single rearing by pooling PSGs from CSR2XCSR4 strain of *Bombyx mori.*

## Data Availability

The data described in this Data note can be freely and openly accessed on NCBI, in the Sequence Read Archive under the accessions and identifiers: *SRX9472457 *(https://identifiers.org/ncbi/insdc.sra:SRX9472457) for day 1, *SRX7730613 *(https://identifiers.org/ncbi/insdc.sra:SRX7730613) for day 5 *and SRX9495290 *(https://identifiers.org/ncbi/insdc.sra:SRX9495290) for day 7 data sets. The other data files are available at the figshare repository: *Data file 1 *(https://doi.org/10.6084/m9.figshare.16760422.v1),* Data file 2: *(https://doi.org/10.6084/m9.figshare.16569543.v2). Please see Table 1 and references [10–13] and [14] for details and links to the data.

## Abbreviations

NuMat: Nuclear matrix
PSG: Posterior silk gland
*B. mori*: *Bombyx mori*
